# Inequalities in care for Iranian women suffering from the comorbidity of substance use and mental illness: The need for integrated treatment

**DOI:** 10.34172/hpp.2023.24

**Published:** 2023-09-11

**Authors:** Mohammad Taghi Kheirkhah, Mehran Mokarrami, Maryam Kazemitabar, Danilo Garcia

**Affiliations:** ^1^Institute for Cognitive and Brain Sciences, Shahid Beheshti University, Tehran, Iran; ^2^Faculty of Psychology and Education, University of Tehran, Tehran, Iran; ^3^Yale School of Medicine, Yale University, New Haven, CT, USA; ^4^VA Connecticut Healthcare System, West Haven, USA; ^5^Department of Behavioral Sciences and Learning, Linköping University, Linköping, Sweden; ^6^Centre for Ethics, Law and Mental Health (CELAM), University of Gothenburg, Gothenburg, Sweden; ^7^Department of Psychology, Lund University, Lund, Sweden

**Keywords:** Health inequities, Mental health services, Substance-related disorders, Women’s health

## Abstract

This paper addresses the comorbidity of substance use and mental illness among women in Iran and the barriers they encounter in accessing treatment. Research has demonstrated a higher prevalence of comorbidity of substance use disorders and mental illness among women than men. It has been suggested that women in Iran may face numerous barriers to appropriate care, such as stigma and discrimination associated with substance use. Integrated treatment for co-occurring disorders (CODs) has been highly beneficial and effective; however, personal and structural limitations impede this treatment approach, which explains the need to develop a situation- and culture-specific program. Needs assessment is necessary to achieve an integrated treatment, and the Iranian government should take the lead in this endeavor. However, if this seems unlikely, non-governmental organizations could be called upon to promote it.

## Introduction

 The comorbidity of substance use and mental illness is a challenging and widespread public health concern, often referred to as co-occurring disorder (COD).^1^ It is critical to understand CODs since they heighten the risk of poor general health, more severe symptoms, suicide, and hesitancy in seeking care.^2^ Substance use can lead to various mental health problems (substance-induced disorders),^3^ or it can be concurrent with pre-existing mental issues. On the other hand, conditions such as anxiety,^4^ depression,^5,6^ attention-deficit/hyperactivity disorder,^7^ etc. can cause substance use disorder (SUD). Research has demonstrated a higher prevalence of comorbidity of SUD and mental illness among women than men,^8^ which may be even more pronounced in countries with broader gender inequalities, such as Middle Eastern countries.^9^ In Iran, substance use is highly stigmatized for women, leading to feelings of shame and embarrassment that discourage them from seeking help.^10^ Furthermore, women in Iran may face numerous barriers to appropriate care, such as preconceived thoughts associated with their substance use, lack of culturally-specific care services, fear of legal repercussions, fear of being ostracized from religious, cultural, and social communities, fear of reprisal from family and community members, negative perceptions of addiction and mental health treatment, traditional values that discourage seeking treatment, discrimination within treatment settings, lack of insurance coverage, economic constraints, and a lack of authority.^11^ COVID-19 lockdowns have also caused an increase in domestic violence in Iran,^12,13^ which could further complicate the issue for women of low socioeconomic status. Despite the significant number of Iranian women with COD, limited and insufficient therapeutic solutions are available to them,^14^ which may explain why women have a higher relapse rate than men.^15^ Additionally, treatment for dual diagnosis can be expensive, and many Iranian women may not have the required financial resources for care, particularly if they are not insured. All these highlight the cultural and structural complexities and challenges that Iranian women encounter when seeking care for substance use disorders. This emphasizes the necessity for culturally sensitive approaches to address these issues.

## Integrated treatment of COD

 In the past, treatment programs for CODs were structured in a way that addressed mental illness and substance use separately. It was common practice to treat each aspect of the problem sequentially prior to the 1990s.^16^ In this traditional approach, patients received therapy for one condition before being permitted to start therapy for the other; however, this technique had a low clinical success rate and significant relapse probability.^17^ In recent decades, there have been developments in COD treatments, including parallel treatment. In parallel treatment, COD patients under substance use treatment are assigned to external mental health care. This entails both mental health and substance use disorder services being received simultaneously but from distinct providers and/or institutions, with no established protocols for care coordination or professional collaboration. Unfortunately, patients have a high relapse rate after being discharged, necessitating them to continue with outpatient substance use treatment while also undergoing mental health services.^18^

 Although there is evidence that dual treatments result in satisfactory outcomes in Iranian women,^19^ there is still an urgent need to develop comprehensive and holistic treatment approaches, including integrated treatment programs, to address the barriers they face in overcoming this challenging and chronic condition. Integrated treatments involve comprehensive services for substance use and mental illnesses provided by a coordinated professional team in the same physical location.^3^ Integrated treatments for individuals with COD have been found to be highly beneficial and effective.^20-24^ Engagement (pre-contemplation), persuasion or motivational intervention (contemplation and preparation), active treatment (action), and maintaining recovery-relapse prevention (maintenance) are identified as crucial stages for COD integrated treatment programs.^25^ Engagement is the initial treatment stage. It involves various aspects such as attracting patients to the program and keeping them engaged. During this stage, the therapeutic goal is to get the patients to accept the problematic nature and negative aspects of their disorder, to assess their self-monitoring and self-regulating capabilities, and to raise their awareness about their condition in general. The next stage of treatment is persuasion or motivational intervention. The aim is to assist patients in making a genuine commitment to treatment by equipping them with the ability to set their own goals to improve their substance use and mental health. The third stage of COD treatment is active treatment or action to manage mental symptoms, attain sobriety, and sustain it. During this stage, treatment programs use various approaches for mental health treatment, such as cognitive-behavioral therapy, family therapy, group therapy, or combinations of multiple approaches. Patients taking medications should receive psychoeducation about medication management, including interactions between the substance and psychotherapeutic medication, as part of their mental health care at this point. Lastly, individuals in recovery strive to maintain their progress while adapting it to their daily life (for a review see Mueser et al^17^).

 Despite the usefulness and effectiveness of integrated treatments, personal and structural limitations such as service availability, appropriate identification of COD, training of providers, provision of services, treatment disparities based on gender, race, and ethnicity, and insurance and policy barriers impede this treatment approach.^26^ While highly competent and adaptable multidisciplinary teams of clinicians may not be available to many communities’ mental health centers, strong leadership, visioning, education, and communication can help foster interdisciplinary teamwork in order to provide efficient integrated solutions. Reliable and consistent facility personnel, constant clinical monitoring and supervision, and program evaluation are also essential for effective integration services.^27^ To overcome personal limitations, it is crucial for patients and their families to be educated and aware of the treatments provided by clinicians, as this can support them during the therapy stages and inform them about the advantages and benefits of integrated treatment services.^22^

## Barriers, needs, and care

 Even though an integrated treatment for Iranian women with COD has its limitations, barriers to receiving care and special therapeutic needs are not yet fully understood. Therefore, identifying the barriers, assessing needs, and overcoming the personal and structural limitations in a treatment program must be investigated. To this end, various tools and instruments such as questionnaires, interviews, focus groups, document analyses, or mapping of cases against evaluation frameworks have been devised and used in quantitative, qualitative, and mixed-method research approaches.^28^ A needs assessment can help determine and address the gaps between the current and desired conditions concerning the phenomena under study; hence, it would be beneficial to improve and enhance the discovered deficiencies. However, needs assessments performed in Iran among women with COD are scarce and limited. Therefore, it is necessary for the first step toward achieving an integrated treatment. Following this, a treatment protocol integrating SUDs and mental illnesses must be developed and examined for Iranian women with COD. It is essential to consider the feasibility, acceptability, and accessibility of the treatment for all parties (i.e., patients, service providers, and the government). It is expected that advancement in this line of study will be helpful for substance use treatment providers to choose a therapeutic solution based on the patient’s specific condition and prerequisites so that the patient and her family can engage in an effective and sustainable treatment and relapse prevention. This will also contribute to expanding the growing literature on COD treatment among Middle Eastern women with new cultural solutions.

## Call to action

 Given the inequities and numerous other barriers rooted in a culture that hinders support for COD among Iranian women, an integrated treatment approach is essential. To ensure the success of such a program, it is necessary to identify the barriers that impede access to appropriate care, as well as the needs related to SUD and mental illness that require an integrated approach.

 The Iranian government’s approach to women suffering from substance use disorders is inadequate and problematic. Women, specifically those with low socioeconomic status in Iran, are often not provided access to the necessary treatment and resources needed to overcome their substance use problems.^11^ Furthermore, women who become addicted to drugs are often harshly judged and stigmatized,^9^ which can lead to feelings of shame and isolation. Additionally, women in Iran are not typically given the same facilities and protections as men regarding drug-related issues, such as sufficient access to treatment programs.^29^ The Iranian government should take a more progressive approach to the issue of women’s CODs, and provide women with the necessary resources and support they need to overcome their problem. Educating society is also essential to change the common culture and reduce stigma. All these are made possible by a situation- and culture-specific integrated treatment approach. The Iranian government should take the lead in this endeavor, but if this seems unlikely, non-governmental organizations could be called upon to promote it.

## Implications for practice

 Recognizing the barriers and needs leads to a deeper understanding of the quality of the problem. Through an integrated approach, practitioners from different disciplines can work together to provide comprehensive care for Iranian women with CODs. By adopting an integrated approach, it will be feasible for practitioners to implement a systematic intervention within the healthcare system, ensuring that women with comorbid conditions receive comprehensive care. This may involve establishing collaborative partnerships between substance use treatment providers and mental health professionals, sharing expertise and resources, and coordinating care plans to address both conditions effectively.

## Acknowledgments

 We would like to acknowledge and thank all service providers, researchers, and those who are engaged in the effort to help individuals recover from substance use disorders. We would also like to extend our thanks to Dr. Kaveh Khoshnood for his invaluable comments, which greatly helped improve the progress of this work.

## Competing Interests

 The authors declare that they have no known competing financial interests or personal relationships that could have appeared to influence the work reported in this paper.

## Ethical Approval

 Not applicable.

## Funding

 Neither the research, authorship, nor publication of this work were supported by funding or other sources.
